# Supplementary material for: The Risk Factors of Chronic Pain in Victims of Violence: A Scoping Review
Source: Healthcare (Basel). 2023 Aug 29;11(17):2421. doi: 10.3390/healthcare11172421 (PMC10486711; doi:10.3390/healthcare11172421)
Supplement: Supplementary file 1 [file healthcare-11-02421-s001.zip › healthcare-2538709-supplementary.pdf]

Summary of quality assessment

Cohort studies

Study	Similarity of the two groups	Similarity of the exposureas measure	Validity and reliability of exposure measur	Identified confounding factors	Strategies to deal with confounding factors	Freedom from the outcome at the start of the study	Validity and reliability of outcomes measure	Follow up time	Completion of follow-up	Strategies to address incomplete follow-up	Appropriateness of statistical analysis
Y-kzv2011	+	+	+	+	?	-	+	-	+	-	+
F-owy2022	+	+	+	+	+	-	+	?	?	?	+

Case-control studies

Study	Comparability of disease or in the absence of this	Appropriate match of cases and controls	Used of same criteria for identification of cases and controls	Validity and reliability of exposure measure	Similarity of the exposure measure	Identified confounding factors	Strategies to deal with confounding factors	Validity and reliability of outcome measure	Exposure period time	Appropriateness of statistical analysis
G-lso2018	+	+	+	?	+	+	+	?	?	+
G-qtr-2019	+	+	+	+	+	?	?	+	+	+
E-mbz2011	+	+	+	+	+	?	?	-	+	+
W-hjp2010	+	+	+	+	+	?	?	+	+	+

Cross-sectional studies

Study	Clear definition of inclusion criteria	Detailed description of the sample and the setting	Validity and reliability of exposure measure	Objectiveness of condition measurement	Identified confounding factors	Strategies to deal with confounding factors	Validity and reliability of outcome measure	Appropriateness of statistical analysis
P-tdz2004	?	-	?	+	+	+	+	+
H-lnb2008	+	+	+	?	-	-	+	+
K-xim2011	+	+	+	?	-	-	+	+
B-ang2007	+	+	+	+	?	?	+	+
V-cse2010	+	+	+	+	?	?	+	+
G-jfo2012	+	-	?	+	+	+	+	+
S-tre2013	+	+	+	+	-	-	+	+
G-yqw2016	+	-	+	+	+	+	+	?
C-dte2010	+	+	+	+	+	?	+	+
E-msn2018	+	+	+	+	+	-	+	+
S-hig2013	+	?	+	+	-	-	+	+
H-uve2017	?	?	+	+	+	+	+	?
G-skc2018	+	+	+	?	-	-	+	+
L-qua2017	+	+	+	+	?	?	+	+
J-scu2020	+	?	+	+	+	-	+	+
A-mia2021	?	+	+	?	+	+	+	+
A-scia2021	+	?	+	?	+	+	+	+
R-phl2021	+	+	+	?	+	-	+	+
D-wgn2021	+	+	+	+	+	?	+	+
